# Spherical nucleic acids as an infectious disease vaccine platform

**DOI:** 10.1073/pnas.2119093119

**Published:** 2022-03-21

**Authors:** Michelle H. Teplensky, Max E. Distler, Caroline D. Kusmierz, Michael Evangelopoulos, Haley Gula, Derek Elli, Anastasia Tomatsidou, Vlad Nicolaescu, Ian Gelarden, Anjana Yeldandi, Daniel Batlle, Dominique Missiakas, Chad A. Mirkin

**Affiliations:** ^a^Department of Chemistry, Northwestern University, Evanston, IL 60208;; ^b^International Institute for Nanotechnology, Northwestern University, Evanston, IL 60208;; ^c^Department of Biomedical Engineering, Northwestern University, Evanston, IL 60208;; ^d^Howard T. Ricketts Laboratory, Department of Microbiology, University of Chicago, Chicago, IL 60637;; ^e^Department of Pathology, Northwestern University Feinberg School of Medicine, Chicago, IL 60611;; ^f^Division of Nephrology and Hypertension, Department of Medicine, Northwestern University Feinberg School of Medicine, Chicago, IL 60611

**Keywords:** antiviral vaccines, spherical nucleic acids, rational vaccinology, infectious disease

## Abstract

Despite recent efforts demonstrating that organization and presentation of vaccine components are just as important as composition in dictating vaccine efficacy, antiviral vaccines have long focused solely on the identification of the immunological target. Herein, we describe a study aimed at exploring how vaccine component presentation in the context of spherical nucleic acids (SNAs) can be used to elicit and maximize an antiviral response. Using COVID-19 as a topical example of an infectious disease with an urgent need for rapid vaccine development, we designed an antiviral SNA vaccine, encapsulating the receptor-binding domain (RBD) subunit into a liposome and decorating the core with a dense shell of CpG motif toll-like receptor 9 agonist oligonucleotides. This vaccine induces memory B cell formation in human cells, and in vivo administration into mice generates robust binding and neutralizing antibody titers. Moreover, the SNA vaccine outperforms multiple simple mixtures incorporating clinically employed adjuvants. Through modular changes to SNA structure, we uncover key relationships and proteomic insights between adjuvant and antigen ratios, concepts potentially translatable across vaccine platforms and disease models. Importantly, when humanized ACE2 transgenic mice were challenged in vivo against a lethal live virus, only mice that received the SNA vaccine had a 100% survival rate and lungs that were clear of virus by plaque analysis. This work underscores the potential for SNAs to be implemented as an easily adaptable and generalizable platform to fight infectious disease and demonstrates the importance of structure and presentation in the design of next-generation antiviral vaccines.

## Significance

Using SARS-CoV-2 as a relevant case study for infectious disease, we investigate the structure–function relationships that dictate antiviral spherical nucleic acid (SNA) vaccine efficacy. We show that the SNA architecture can be rapidly employed to target COVID-19 through incorporation of the receptor-binding domain, and that the resulting vaccine potently activates human cells in vitro and mice in vivo. Furthermore, when challenged with a lethal viral infection, only mice treated with the SNA vaccine survived. Taken together, this work underscores the importance of rational vaccine design for infectious disease to yield vaccines that elicit more potent immune responses to effectively fight disease.

Infectious diseases have long threatened humanity due to their ability to rapidly spread and mutate across populations, infecting many people (1). The rapid and global spread of severe acute respiratory syndrome coronavirus 2 (SARS-CoV-2), the virus that causes COVID-19, emphatically revealed this and highlighted the importance of effective vaccination strategies to mitigate the spread and infectivity of viruses. Vaccination strategies are increasingly important as we consider the potential for emerging infectious diseases still to come (2, 3). The ability to rapidly adapt vaccine platforms through advancement of previous knowledge can be a huge asset. In particular, protein-based subunit vaccines can reduce vaccine production costs, while diminishing vaccine side effects. However, ultimate outcomes of protein-based subunit vaccine performance are difficult to correlate between candidates (4).

An example of this is the influenza vaccine, which has relied on various simple mixtures of antigenic protein subunit target and adjuvant in solution to induce immune responses (5). As a result, influenza vaccine effectiveness has varied dramatically by year, with a low of 10% effectiveness in 2004–2005 and a high of 60% effectiveness in 2010–2011 (6, 7). This high variability is often attributed to the level of antigenic match between circulating viruses and vaccine strains. However, recent work has shown that the same antigen target can be more or less antigenic depending on the mode of presentation and delivery to the immune system (7, 8). By harnessing this concept, which we have termed rational vaccinology (9), we can greatly aid efforts to correlate vaccine design with performance by providing structurally informed and optimized vaccine platforms that can be readily and quickly adapted to new disease targets.

Rational vaccinology has been implemented successfully for vaccines against cancer, where nanoscale changes have dramatically altered immune activation and tumor reduction (9–11). The application of this approach toward infectious disease has yet to be fully realized, and the potential for it to dramatically impact the success of vaccine development remains untapped. Herein, we have implemented spherical nucleic acid (SNA) nanotechnology as a tool to explore the impact of vaccine presentation when applied to infectious disease, using COVID-19 as a case study. SNAs comprise a nanoparticle core surrounded by a dense radial arrangement of oligonucleotides (12–14). Like many nanovaccine platforms, the SNA is biocompatible and comprises naturally found molecules in cellular biology. Importantly, however, the SNA provides key advantages over other nanovaccine platforms. Specifically, the SNA platform is highly modular, enabling the elucidation of important structure–function relationships. Moreover, the SNA is effective at entering cells rapidly and in high quantities through scavenger receptor A–mediated endocytosis and is resistant to nuclease degradation, due to the dense arrangement of oligonucleotides (15, 16). Moreover, by using a DNA shell containing immunostimulatory CpG motif DNA, SNAs robustly activate the innate immune system through toll-like receptor 9 (TLR9) (9, 17) and exhibit efficient lymph node drainage and high codelivery of adjuvant and antigen to antigen-presenting cells (9, 11). These properties have been harnessed in this work to maximize humoral responses and generate antibodies that are effective at neutralization in pseudoviral assays, capable of withstanding mutations to still bind the target, and protective in mice against a lethal viral challenge. Overall, we report enhancement in immune responses, leading to a 100% survival rate in a lethal viral challenge, which can be achieved through utilization of the SNA’s privileged architecture.

## Results

### SNA Design and Characterization.

**Fig. 1.**
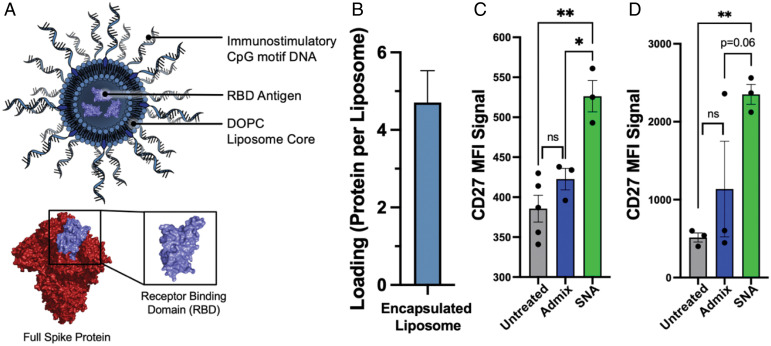
An SNA vaccine containing RBD antigen is capable of activating B cells in vitro. (*A*) (*Top*) A schematic of the SNA used in this work encapsulating RBD antigen within an 80-nm DOPC liposome core, and radially displaying a shell of TLR9-agonist CpG motif DNA. (*Bottom*) RBD structure (purple) as a subset of the full SARS-CoV-2 Spike protein (red). This representation of the full Spike protein and RBD was adapted from Protein Data Bank ID 6VXX. (*B*) Minimal batch-to-batch variation of the amount of RBD protein loaded per liposome (mean and 95% CI shown, *n* = 10). (*C* and *D*) In vitro activation of hPBMCs to increase expression of CD27 among CD19^+^ B cells. Incubation with hPBMCs was performed for both 1 d (*C*) and 3 d (*D*). Mean and SEM are shown; analysis was done using an ordinary one-way ANOVA followed by a (*C*) Sidak’s or (*D*) Dunnett’s multiple comparisons test; *n* = 3 to 4 per group. *P < 0.05; **P < 0.01; other *P* values are shown.

To synthesize SNA vaccines capable of raising robust, prophylactic responses against SARS-CoV-2, we harnessed the modularity of the liposomal SNA to simultaneously deliver encapsulated protein antigen and CpG motif DNA adjuvant. The modularity of the SNA platform enables fine-tuned control over vaccine structure, allowing for the rational design of the most effective vaccine. For these studies, we used the receptor-binding domain (RBD; *SI Appendix*, Fig. S1) of the SARS-CoV-2 Spike protein as the antigen, because this domain is responsible for recognizing and binding to human cell’s angiotensin-converting enzyme 2 (ACE2) receptor and facilitating cellular entry. SNAs were synthesized using previously established protocols (9, 18). Briefly, protein antigens were encapsulated in 80-nm liposomes prepared from 1,2-dioleoyl-sn-glycero-3-phosphocholine (DOPC) films and purified using tangential flow filtration (TFF) to remove any unencapsulated protein. To form SNAs, protein-encapsulated liposomes were incubated with 3′-cholesterol-modified CpG DNA (Fig. 1*A*). The CpG DNA used was either a human or murine TLR9 agonist sequence, depending on the experiment (*SI Appendix*, Table S1). Successful DNA incorporation and SNA formation was confirmed by dynamic light scattering (DLS) and agarose gel electrophoresis, which shows a decrease in electrophoretic mobility commensurate with DNA loading and increased size (*SI Appendix*, Fig. S2). The average RBD protein loading per liposome across 10 distinct batches was 4.7 ± 1.6 (Fig. 1*B*). This is equivalent to a loading capacity of 0.52 wt/wt %.

### B Cell Activation In Vitro in Human Peripheral Blood Mononuclear Cells.

A necessary step in effective vaccination is immunological memory carried by memory B cells, as these are easily reactivated upon exposure to antigen (19–21). Moreover, their activation results in rapid proliferation and differentiation into plasma cells that produce large amounts of higher-affinity antibodies (19, 20). SNAs were therefore assessed for their ability to robustly activate naïve B cells in human peripheral blood mononuclear cells (hPBMCs). For these studies, we used SNAs composed of a human CpG 7909 oligonucleotide shell (*SI Appendix*, Table S1). After 1 and 3 d of incubation, cells were measured for the presence of CD27, an activation marker which can contribute to B cell expansion, differentiation, and antibody production (22, 23). A significant difference in CD27 expression was observed when comparing SNA to simple mixtures of RBD and CpG, termed admix, and when compared to untreated cells (Fig. 1 *C* and *D*). This can be attributed to the advantageous properties that emerge when utilizing the SNA architecture, such as improved codelivery of antigen and adjuvant components, increased and rapid cellular uptake, and enhanced resistance to nuclease degradation (9, 11, 24). While using human cells, this demonstration of B cell activation is in vitro, and does not consider the complexity involved in multicell cross-talk that leads to robust antibody production. Therefore, we next assessed the ability of the SNA to stimulate the adaptive immune system in vivo to generate robust, antigen-specific antibody responses.

### In Vivo Antibody Production.

**Fig. 2.**
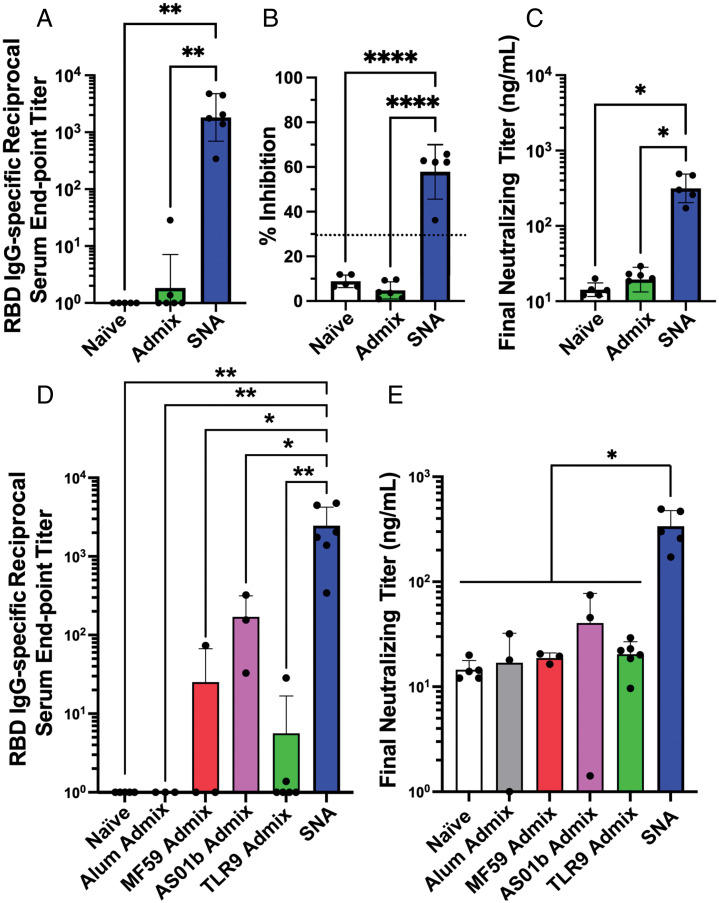
SNA structure induces higher levels of antigen-specific binding and neutralizing titers in vivo. (*A*) Samples were quantified via ELISA for the presence of RBD-specific IgG binding antibodies. Reciprocal serum end point titers were calculated through fitting absorbance at 450-nm values to a four-parameter logistic sigmoidal curve. (*B*) Sera were employed in a pseudovirus inhibition study and were assessed for the inhibition percentage at a 1:10 sera dilution, or (*C*) were fit to a standard curve to calculate a final neutralizing titer. Dashed line in *B* represents threshold cutoff value for positive inhibition according to manufacturer’s protocol. (*D*) RBD-specific IgG binding antibody measurement of SNA compared with simple mixture immunizations formulated using commercial adjuvants. (*E*) Final neutralizing titer calculated in a pseudovirus inhibition study and fit to a standard curve. All graphs show mean and SD, *n* = 3 to 6 per group. Mice were injected with 1.4 nmol by RBD protein and one of the following adjuvants: 44 nmol by CpG DNA (SNA and TLR9 admix groups), 40 μg by Al^3+^ (Alum admix), 25 μL by AddaVax (MF59 admix), or 4.2 μg by QS21 and MPLA4 (AS01b admix). Dosing can be found in greater detail in *In Vivo Immunization in Mice.* For *A, B,* and *D*, analysis was done using an ordinary one-way ANOVA followed by a Tukey’s multiple comparisons test. For *C* and *E*, analysis was done using a Brown–Forsythe ANOVA followed by a Dunnett’s multiple comparisons test. *P < 0.05; **P < 0.01; ****P < 0.0001.

We evaluated RBD IgG-specific binding and neutralizing antibody production following a single subcutaneous injection in C57BL/6 mice (*n* = 3 to 6 per group). RBD-specific binding antibodies were assessed by ELISA on mouse sera collected 2 wk postprime injection. For SNA-immunized mice, we quantified a *ca.* 1,000-fold enhancement in the reciprocal serum end point antibody titer compared to the simple mixture control (Fig. 2*A*). Furthermore, SNA-treated mice elicited a potent pseudovirus-neutralizing ability, whereby the generated antibodies inhibited 58% of the interaction between RBD and ACE2 at a 1:10 dilution in a surrogate virus neutralization test (sVNT) assay (Fig. 2*B*). Overall, a *ca.* 16.5-fold enhancement of final neutralizing antibody titer was measured for SNA-treated mice compared to those that received admix (Fig. 2*C*), which had no detectable inhibition ability (threshold was 30% inhibition as per manufacturer’s specifications) (Fig. 2*B*).

These assays validate that serum antibodies are more robustly produced with SNA immunization compared to admix vaccination, and also that a single dose of the SNA vaccine generates antibodies that can recognize and block a pseudoviral receptor binding domain from binding to an ACE2 receptor. This underscores the importance of structuring components into an SNA architecture in order to achieve enhanced biological and therapeutic responses, as proteins and DNA alone exhibit poor biodistribution to draining lymph nodes and rapid clearance (25). By contrast, these results uphold that SNAs effectively deliver cargo to immune cells in vivo. Moreover, high neutralizing antibody levels are correlated with protection against infection (26); therefore, these results establish the potential for SNA vaccines to be a viable vaccination strategy for COVID-19.

### Comparison of SNA Performance to Simple Mixtures of RBD and Commercial Adjuvants.

To further compare the SNA platform against commercially available alternatives (27), we evaluated how the SNA vaccine compared against mixtures of commercial clinically used adjuvants and the RBD antigen. Specifically, we employed Alum, MF59, and AS01b as adjuvants. All of these have been used to protect against infectious diseases, including hepatitis B or influenza (28–30). In an assessment of RBD IgG-specific binding antibodies 2 wk postprime, SNA treatment outperformed all tested simple mixtures of adjuvants, even surpassing the best-performing commercial adjuvant AS01b simple mixture by 14-fold. It ultimately reached a reciprocal serum end point titer of 2,464 (Fig. 2*D*). The simple mixture formulated with alum admix has undetectable binding antibodies indistinguishable from those raised by naïve mice, and MF59 admix had only one responder out of three mice, which had a reciprocal serum end point titer of 74, 33-fold lower than SNA vaccination. When comparing SNA against commercial adjuvant-containing simple mixtures in a pseudoviral neutralizing study (Fig. 2*E*), even the best-performing group, AS01b admix, only reached 41 ng/mL neutralizing antibodies in sera, whereas the SNA concentration is nearly ninefold higher, 339 ng/mL. This trend was similarly observed 3 wk postprime (*SI Appendix*, Fig. S3), where the sera neutralizing antibodies generated from the SNA immunization were highest at 1,193 ng/mL, fourfold greater than the closest admix (AS01b).

### Stoichiometry between Adjuvant and Antigen Impacts Immune Response.

**Fig. 3.**
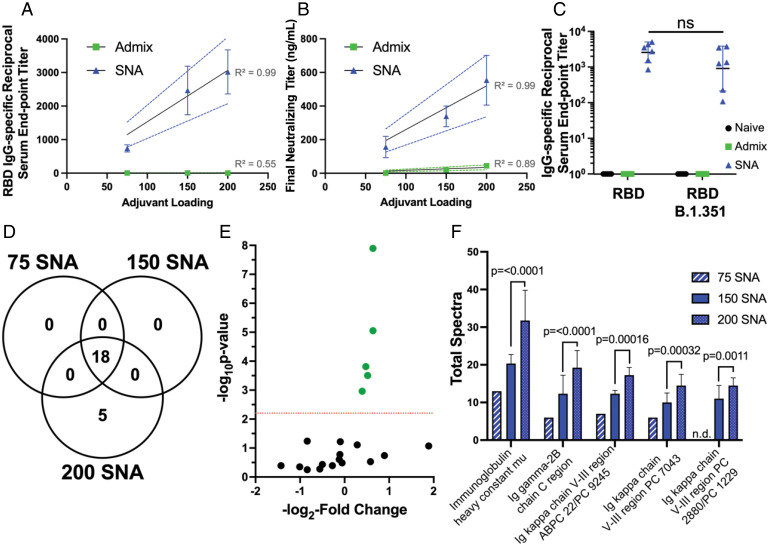
SNA vaccines formulated with different loadings of adjuvant DNA as the shell demonstrate linear correlations in resulting binding and neutralizing antibody production as a result of differential protein expression. (*A*) RBD-specific IgG binding antibodies shown as the reciprocal serum end point titer were plotted against adjuvant loading for the three different SNA groups. (*B*) The pseudovirus inhibition assay demonstrated the same positive linear relationship between adjuvant loading and the calculated final neutralizing titer. Graphs show mean and SEM for *n* = 3 to 6 per group. (*C*) Antibodies generated by the different vaccines were assessed for their ability to bind the B.1.351 variant of the RBD protein. Graph shows mean and SD for *n* = 3 to 6 per group. Analysis was performed doing a two-way ANOVA followed by Sidak’s multiple comparisons test; ns denotes nonsignificant change in reciprocal serum end point titer for SNA-raised antibodies. (*D*) Quantitative profile of Igs between the three SNA groups with different loadings of adjuvant DNA on the shell. (*E*) Volcano plot showing relative fold change and significance of different Igs when comparing the 200-SNA against the 150-SNA group. Red line indicates significance threshold. (*F*) The five identified up-regulated proteins were plotted as a function of total spectra with significance between 200 SNA versus 150 SNA shown; n.d. denotes not detected. Significance threshold is *P* < 0.0063. Mice were injected with the following: SNA 200 and admix equivalent were dosed at 1.4 nmol by RBD protein, and 60 nmol by CpG DNA. SNA 150 and admix equivalent was dosed at 1.4 nmol by RBD protein, and 44 nmol by CpG DNA. SNA 75 and admix equivalent was dosed at 1.4 nmol by RBD protein, and 22 nmol by CpG DNA. Dosing can be found in greater detail in *In Vivo Immunization in Mice*.

Utilizing the modularity of the SNA architecture, we sought to understand the impact of the amount of adjuvant DNA loading on the SNA shell. We performed an agarose gel on 80-nm DOPC liposomes to evaluate the range of DNA that could fit on the surface before dissociating (31) (*SI Appendix*, Fig. S4). This revealed a maximum of *ca.* 200 DNA strands per liposome (surface density of 1.7 pmol/cm^2^), which aligns with other liposomal SNA structures (10, 32). To investigate the adjuvant loading dependence on vaccine efficacy, we varied the DNA surface coverage to synthesize three different SNAs containing 75, 150, and 200 strands per liposome, while keeping the encapsulation of protein in the core constant; this provided three different adjuvant:antigen ratios that were *ca.* 16:1, 32:1, and 43:1, respectively. We hypothesized that enhanced loading would propagate an initial innate response, which could enhance an adaptive response. Mice were inoculated with one of the three different SNAs or one of three TLR9 admix controls that matched these adjuvant:antigen ratios. Two weeks postprime injection, RBD-specific binding and neutralizing antibodies were quantified from sera and were plotted against adjuvant loading to determine any linear relationships (Fig. 3 *A* and *B*). There is a strong positive correlation between adjuvant loading and immune response for SNA immunization (*R*^2^ = 0.99 for both binding and neutralizing antibodies). Moreover, we assessed the ability of antibodies generated from the best-performing SNA (200 adjuvant strands per liposome) to bind to a mutated RBD of an evasive variant, B.1.351 (33). There was a nonsignificant difference in the reciprocal serum end point titer when binding to the RBD B.1.351 variant (Fig. 3*C*), when compared to the reciprocal serum end point titer for binding to wild-type RBD. This promising retention of antibody binding ability leads us to propose that the SNA platform can generate robust humoral responses that are resistant to mutational viral changes.

### Identification of Up-Regulated Immunoglobulin Classes Using Proteomics.

To understand how the different stoichiometries of adjuvant on SNA induced different levels of binding and neutralizing antibodies, we performed proteomics to assess the expression of different immunoglobulins (Igs). Eighteen Igs were identified among all of the SNA formulations (75, 150, or 200 adjuvant strands per liposome termed 75 SNA, 150 SNA, and 200 SNA, respectively), with an additional five distinct Ig proteins present in the 200-SNA treatment group (Fig. 3*D*). In particular, the five significant proteins have increased fold changes in the 200-SNA immunization compared to 150 SNA (Fig. 3*E*). Furthermore, the total spectra of these proteins, an indicator of protein abundance (34), increases as the adjuvant loading increases (Fig. 3*F*). The IgM antibody was one of the 18 Ig proteins present among all SNA vaccine groups (*SI Appendix*, Table S2) and is the first Ig class produced in the primary response to antigens (35). The five significant Igs in the 200-SNA group are involved in antigen binding, positive regulation of B cell activation and B cell receptor signaling, Ig receptor binding and mediated immune response, and, overall, the adaptive immune response (36). This suggests that the increased expression of these proteins and the subsequent processes in which they are involved result in the measurable increase in immune outputs.

### Dosing of SNA Vaccine Enhances Immune Responses.

**Fig. 4.**
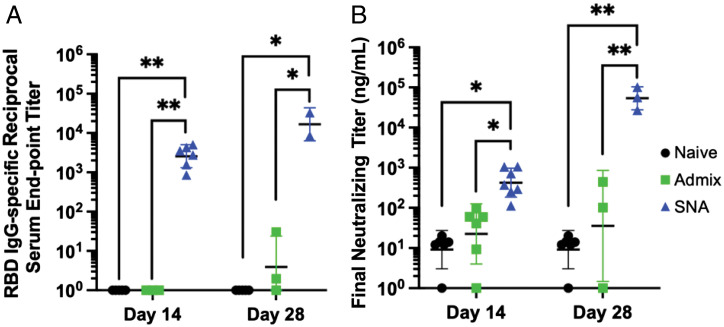
Prime–boost vaccination with two administrations enhances antibody production for all treatments, while SNA immunization is highest. Levels of (*A*) binding and (*B*) neutralizing antibodies on either day 14 (prime only on day 0, sera collection on day 14) or on day 28 (prime on day 0, boost on day 14, sera collection on day 28). Graphs show mean and SD for *n* = 3 to 6 per group. Analysis was done using a two-way ANOVA followed by a Tukey’s multiple comparisons test. Only significant comparisons are shown. *P < 0.05; **P < 0.01.

We quantified the impact of multiple doses of the best-performing SNA vaccine (200 SNA) on the resulting amplification of immune responses. Many ongoing vaccines that were granted emergency use authorization or are in development have utilized a prime–boost vaccine schedule to enhance immune responses (37). Therefore, we immunized mice with a prime–boost schedule (boost was 2 wk postprime) to assess any elevation in antibody production. We collected sera from the mice 2 wk after the boost, which is 4 wk (28 d) after the initial prime immunization. An increase in both binding antibodies (Fig. 4*A*; 6.5-fold) and neutralizing antibodies (Fig. 4*B*; 126-fold) was observed when comparing sera from two doses to that from one. The admix vaccination did improve with a second administration, but levels of binding and neutralizing antibodies were still 4,000-fold and 1,500-fold lower, respectively, compared to the SNA immunization (Fig. 4), and proteomics signatures were significantly different (*SI Appendix*, Fig. S5).

### Live Viral In Vivo Challenge Using Transgenic k18-hACE2 Mice.

**Fig. 5.**
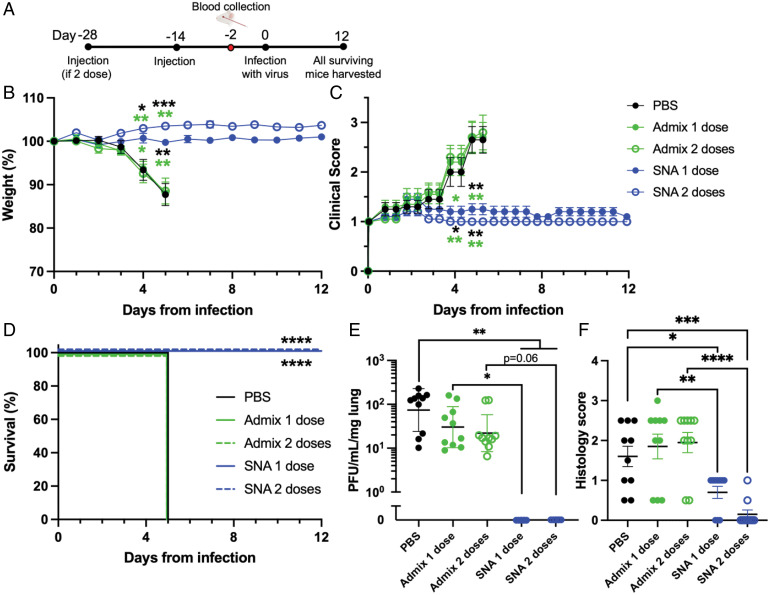
Vaccine effectiveness was tested using k18-hACE2 transgenic mice in a live viral SARS-CoV-2 challenge study. (*A*) Female and male mice (*n* = 10 total per group, 5 of each gender) were treated with vaccine or used as control groups and infected with virus as per the schedule. (*B*) Vaccination with SNA of either one or two doses prevented any body weight loss, (*C*) improved clinical scores, and (*D*) prevented any mortality as compared to vehicle mice (PBS) or admix mice treated with one or two doses when infected with SARS-CoV-2. On the date of death, lungs were harvested and assessed for (*E*) viral load and (*F*) histopathology. (*E*) No detectable virus was observed for SNA mice treated with one or two doses. (*F*) Scores of neutrophil infiltration were lower in mice treated with SNA vaccine compared to mice treated with admix vaccine or untreated. Comparisons were made between PBS and all other groups, and admix 1 dose versus SNA 1 dose, and admix 2 dose versus SNA 2 dose. For *B* and *C*, statistical significance is shown above the date at which the analysis was performed. Colors correspond to the group that SNA was compared to. Only significant comparisons were shown, and comparisons were made between SNA and PBS or the admix group with the same corresponding number of doses. For *B*, *C,* and *E*, analysis was done using a Brown–Forsythe ANOVA followed by a Dunnett’s multiple comparisons test. For *F*, analysis was done using an ordinary one-way ANOVA followed by Sidak’s multiple comparisons test. *D* was analyzed using a log-rank test. *P < 0.05; **P < 0.01; ***P < 0.001; ****P < 0.0001.

As a direct test of vaccine efficacy, we conducted a viral challenge study using transgenic mice that are susceptible to infection through expression of the human ACE2 protein: k18-hACE2 (38, 39). Animals were challenged with a lethal dose of the virus (40–44). We compared the top-performing vaccine design (200 SNA) to the admix vaccine, and also to mice receiving only saline (phosphate-buffered saline [PBS]) as a negative control. Mice were given either one or two doses of SNA or admix vaccine. Mice (*n* = 10 per group, comprising 5 females and 5 males) were challenged with virus 2 wk after receiving the final vaccine dose (complete schedule in Fig. 5*A*). Just prior to viral infection, blood was collected from the mice to verify neutralizing antibody production (*SI Appendix*, Fig. S6). After viral infection, animals were monitored twice daily for signs of disease, and weighed daily. Within the first 5 d postinfection, mice that received either saline or one or two doses of admix vaccine experienced a rapid decline in body weight and an increase in clinical score, both of which indicate that the mice were not protected from the virus (Fig. 5 *B* and *C*). These animals were killed humanely at day 5, as per study protocol, and lungs were removed to measure viral loads (Fig. 5 *D* and *E*). In stark contrast, mice that were treated with either one or two doses of the SNA vaccine displayed no evidence of declining health. Body weight and clinical scores remained stable throughout the entire study, and thus survival of the SNA-treated mice was 100% (Fig. 5 *B*–*D*). All surviving animals were killed at day 12 postinfection. Lungs from killed mice (day 5 for PBS and admix vaccine either dose, and day 12 for SNA vaccine either dose) were collected and quantified for viral titers measured by plaque assay. Mice receiving either one or two doses of SNA vaccine had no measurable viral titers in the lungs (Fig. 5*E*). Mice immunized with PBS or either admix dose and killed on day 5, had viral titers around 7 × 10^1^ plaque-forming unit (pfu)/mL/mg lung or 2 to 3 × 10^1^ pfu/mL/mg lung, respectively. Histopathological examination of the lungs, performed following methods used in the same transgenic model (40), showed extensive neutrophil infiltration for mice that did not receive the SNA vaccine (mean = 1.6, 1.85, and 1.95 for PBS-treated, admix 1, and admix 2 dose treated mice, respectively) (Fig. 5*F*). In mice that received the SNA vaccine, neutrophil infiltration was reduced (mean = 0.7 and 0.15 for one and two doses, respectively). This indicates that the SNA vaccine is capable of avoiding or greatly attenuating outcomes of severe COVID-19 pneumonia, which is characterized by elevated neutrophil infiltration (45). Additional histopathological analysis and representative images can be found in *SI Appendix*, Fig. S7. Overall, these results emphasize the impact that the SNA vaccine platform, and, more broadly, rational vaccinology, can have on elevating antiviral vaccine efficacy.

## Discussion

This work establishes the SNA as an effective platform for antiviral vaccines. By utilizing the highly modular SNA architecture, we highlight the importance of packaging viral antigens to raise humoral immune responses that can effectively fight a live virus. This work has important implications for the design of next-generation infectious disease vaccines. It illustrates that antibody production is tunable through simple chemical adjustments (i.e., the adjuvant loading on a liposome), and that a simple change to the ratio of components can greatly alter Ig expression. This work offers alternative strategies to enhancing antibody responses rather than traditional approaches which involve administering multiple doses. Importantly, we also observed that a traditional approach of supplementing a vaccine with adjuvant to enhance an antibody response is not a consistently effective strategy. The results using the SNA, which is compositionally similar to the AS01b simple mixture in that both involve liposomal constructs, suggest that radial display of CpG adjuvant and codelivery of vaccine components, which the SNA provides, leads to a more effective vaccine that can prevent mortality and attenuate lung injury. Future studies can investigate the dose-dependent nature of vaccine efficacy and compare the SNA architecture to alternative vaccine platforms in clinical trials. Consistently, the three-dimensional arrangement of components on the SNA architecture leads to significant increases in vaccine functionality compared to numerous tested simple mixtures and underscores the important role that rational vaccinology will play in future vaccine design.

Taken together, this work underscores the SNA’s potential to be used as a platform for infectious diseases, and that the concept of rational vaccinology holds equally as true for infectious disease as it does for cancer vaccine applications. Given the SNA’s easily adaptable structure to contain any viral antigen and combinations thereof, modulate positions and tune the stoichiometry of each component, and remain stable at room temperature (RT), the SNA is poised to be a rapidly accessible future platform for targets yet to be discovered. When considering the programmability of the SNA architecture, rapid translation to antigenic variants and human adjuvant sequences is easily feasible. Collectively, these results have broad implications for the development of vaccines for COVID-19 and, potentially, other infectious diseases.

## Materials and Methods

### Materials and Animals.

Unless otherwise noted, all reagents were purchased commercially and used as received. Oligonucleotides were synthesized as described below. Proteins were obtained from Northwestern’s Recombinant Protein Production core. Chemicals were purchased from suppliers listed in parentheses. C57BL/6 female mice, age 8 wk to 12 wk old, and k18-hACE2 male and female mice (stock no. 034860), age 6 wk to 8 wk old, were purchased from Jackson Laboratory. Mice were used in accordance with all national and local guidelines and regulations, and protocols performed were approved by the institutional animal care and use committee (IUCAC) at Northwestern University and University of Chicago. Experiments with SARS-CoV-2 were performed in biosafety level 3 (BSL3) and animal BSL3 containment in accordance with the institutional guidelines following experimental protocol review and approval by the Institutional Biosafety Committee and the IACUC at the University of Chicago.

### RBD Protein Expression.

Proteins were expressed using standard protocols in Expi293 system (Thermo Fisher Scientific). First, a vector for the RBD from SARS-CoV-2 (amino acids 319 to 541) was obtained from BEI Resources (NR-52309). The sequence was designed by fusing the RBD sequence with a C-terminal hexahistidine tag and is intended for pCAGGS mammalian expression under the AG promoter. Successful expression was confirmed by sodium dodecyl sulfate (SDS) polyacrylamide gel electrophoresis (*SI Appendix*, Fig. S1).

### Oligonucleotide Synthesis.

Oligonucleotides (*SI Appendix*, Table S1) were generated using an ABI-394 automated DNA synthesizer using standard phosphoramidite chemistry as has been previously reported (24). The 3′-cholesteryl-TEG CPG solid supports and phosphoramidites were obtained from Glen Research. Sequences were synthesized with a phosphorothioate backbone using 4,5-dicyanoimidazole as an activator and 3-((dimethylamino-methylidene)amino)-3H-1,2,4-dithiazole-3-thione as the sulfurizing agent. Following synthesis, the strands were deprotected following previous methods (24) using a 1:1 solution of 37% ammonium hydroxide/40% methylamine (Sigma) at 55 °C for 35 min. The strands were then purified using a C4 column on reverse phase high-performance liquid chromatography (Shimadzu), using a gradient of Buffer A (0.1 M triethylammonium acetate [Sigma] and 3% acetonitrile [Sigma] in water) to pure acetonitrile over 45 min, and the peaks were collected as fractions. The dimethoxytrityl (DMT) group was removed from the product strands by incubation in 20% aqueous acetic acid (Sigma) at RT for 1 h, followed by three washes with ethyl acetate (Sigma) to remove DMT. The final product was lyophilized and resuspended in deionized water (diH_2_O). The concentration was measured using ultraviolet (UV)-visible absorption at 260 nm with extinction coefficients calculated through the IDT OligoAnalyzer online tool (listed in *SI Appendix*, Table S1). The molecular weight of the sequences was measured by matrix-assisted laser desorption time of flight with a Bruker Rapiflex and compared to calculated molecular weight estimates via the IDT OligoAnalyzer Tool.

### Synthesis of RBD-Encapsulated Liposomal SNAs.

Dried lipid films of 50 mg of DOPC (Avanti Polar Lipids) were hydrated with 1 mL of RBD protein (∼7 mg/mL in Dulbecco’s PBS) and 1.5 mL of PBS. Liposomes were formed following 20 freeze–thaw cycles (liquid nitrogen and sonication in a 37 °C water bath), followed by extrusion to 80 nm. The liposomes were extruded using sequential high-pressure extrusion (Northern Lipids Inc.) using polycarbonate filters with pore sizes of 200, 100, and 80 nm; liposomes were passed through each pore size three times. Following extrusion, the liposomes were concentrated down to ∼2 mL to 3 mL using TFF using a filter with a pore size of 500 kDa (Spectrum). To remove unencapsulated RBD, the solution was either passed through the TFF membrane an additional two times or was dialyzed overnight using a 1,000-kDa molecular weight cutoff membrane against 3.5 L of PBS, depending on time constraints. The liposome concentration was determined using a phosphatidylcholine assay kit (Sigma), assuming an 80-nm liposome contains 49,974 lipids per liposome based on

$$N=17.69\;\times \;\left[{\left(\frac{d}{2}\right)}^{2}+{\left(\frac{d}{2}-5\right)}^{2}\right],$$

where *d* is the diameter of the liposome and *N* is the total number of lipids per liposome.

The amount of RBD encapsulated was measured using a bicinchoninic acid (BCA) assay (Thermo-Fisher) after bursting liposomes with 1% SDS. The loading of protein per liposome was calculated by dividing the protein concentration by the liposome concentration.

To form SNAs, 3′ cholesterol-terminated CpG 1826 or CpG 7909 DNA were added to the liposomes in one of the three oligonucleotide to liposome ratios defined, 75:1, 150:1, or 200:1, depending on the experiment. Solutions were incubated at 37 °C overnight and stored at 4 °C.

### Characterization of RBD-Loaded Liposomal SNAs.

Successful SNA formation was characterized by DLS (Malvern Zetasizer) and visualized by agarose gel electrophoresis. Free linear cyanine 5-fluorophore-labeled DNA (*SI Appendix*, Table S1, CpG 1826 Cy5 Fluorophore-labeled) and formed SNAs were loaded into a 1% agarose gel on ice and run at 70 V for 45 min prior to imaging with a Chemidoc Gel Scanner (BioRad) (*SI Appendix*, Fig. S2).

### In Vitro hPBMC B Cell Activation.

This method was adopted from previously published work (24). Briefly, hPBMCs, obtained from Zenbio (SER-PBMC-200P-F), were thawed from storage in liquid nitrogen in a water bath. Cells were mixed and added to 10 mL of Roswell Park Memorial Institute (RPMI) media containing 10% heat-inactivated fetal bovine serum (HI-FBS) and 1% penicillin–streptomycin (denoted herein as RPMI^+/+^). The solution was centrifuged at 300 × *g* for 10 min to pellet the cells. The supernatant was aspirated, and the cells were resuspended in 4 mL of media and counted using a Vi-CELL BLU Cell Viability Analyzer. Cells were diluted to a concentration of 1 × 10^6^ cells per mL, and 100 μL of cell stock was added to wells in a 96-well round-bottom plate. Respective samples were added to cells in 100-μL volume so that the final concentrations were 5 and 124 nM by RBD protein and CpG 7909 DNA, respectively. After either 1 d or 3 d, samples were transferred to flow inserts and washed with 600 μL of PBS. Tubes were spun at 1,200 rpm for 5 min, after which the supernatant was aspirated, and the samples were stained at 4 °C for 15 min in 100 μL of PBS containing a solution of (0.5 μL of: fixable live/dead antibody-UV, CD19-BV421; HLA-DR- PerCP-Cy5.5; CD27-BV605). Cells were then washed with 600 μL PBS, centrifuged at 1,200 rpm for 5 min, aspirated, and resuspended in 100 μL of fixation buffer (BioLegend). Samples were stored at 4 °C prior to flow cytometry analysis. Cells were analyzed by flow cytometry using a BD FACSymphony flow cytometer, and cell events were gated and analyzed on FlowJo.

### In Vivo Immunization in Mice.

Mice were injected subcutaneously in the abdomen with one of the following treatments diluted in saline. Volume was kept below 200 μL. SNA 200 and simple mixture equivalent were dosed at 1.4 nmol by RBD protein, and 60 nmol by CpG DNA. SNA 150 and simple mixture equivalent were dosed at 1.4 nmol by RBD protein, and 44 nmol by CpG DNA. SNA 75 and simple mixture equivalent were dosed at 1.4 nmol by RBD protein, and 22 nmol by CpG DNA. Alum admix was dosed at 1.4 nmol by RBD protein, and 40 μg by Al^3+^ (46). Alhyrdogel adjuvant 2% was used as the source (obtained from InvivoGen). MF59 admix was dosed at 1.4 nmol by RBD protein, and 25 μL by AddaVax adjuvant (obtained from InvivoGen) (47). AS01b admix was first synthesized as described in the patent WO 96/33739 (48). Briefly, liposomes were made comprising DOPC, cholesterol, and MPLA4 in a ratio of 20:5:1, respectively. These liposomes were added to QS21 (Quil-A, InvivoGen) at a ratio of 1:1 MPLA4:QS21. The dose used was determined by converting the typical human dose to the mouse dose used in Brando et al. (49), 4.2 μg by QS21 and MPLA4.

### Retroorbital Blood Collection.

Two weeks after the injection, blood was collected via a retroorbital blood draw. Animals were anesthetized with isoflurane, and, once asleep, a heparin (Sigma) lined pipette was inserted through the conjunctiva and into the orbital sinus by quickly rotating the tube. Approximately 100 μL of blood was drawn and stored at RT for at least 30 min to allow the blood to clot. The blood was then centrifuged at 1,000 × *g* for 10 min, and the supernatant (serum) was carefully removed and transferred to a 96-well plate for downstream analysis. If not used immediately, serum was stored at −80 °C.

### RBD-Specific IgG Binding Antibodies via ELISA.

A 96-well ELISA Uncoated Plate (BioLegend Nunc Maxi Sorp) was coated with 2.5 μg/mL RBD protein diluted in 5× ELISA Coating Buffer (BioLegend) for 2 h at 37 °C. After coating, the plate was washed and subsequently blocked for 2 h at 37 °C with 200 μL of PBS containing 10% FBS and 0.1% Tween‐20 (Sigma). Mouse sera were diluted to various dilution concentrations (e.g., 50×, 100×, 500×, 1,000×, 2,500×, and/or 5,000×) in 5× ELISA dilution buffer (eBioscience). Upon completion of blocking, the blocking solution was removed, 100 uL of each diluted sample was added immediately to each well, and the plate was incubated for 1 h at 37 °C. Following incubation, the plate was washed three times with PBS containing 0.1% Tween‐20. Then 100 μL of secondary antibody (Goat anti‐mouse IgG‐ horseradish peroxidase [HRP], BioLegend), diluted 1:4,000 in 5× ELISA dilution buffer, was added to each well and incubated at RT for 1 h. The solution was washed out with three cycles of PBS containing 0.1% Tween‐20. A 1:1 mixture of tetramethylbenzidine (TMB) Reagent A and Reagent B (BioLegend) was made and immediately used by adding 100 μL to each well. The plate was incubated at RT in the dark for 3 min, at which point, once the color became pronounced, 100 μL of TMB Stop Solution (BioLegend) was added. The absorbance was immediately read at 450 nm using a BioTek Cytation 5. Titers were defined as the reciprocal serum dilution where 450-nm absorbance was at least 0.5 units above the background sample (i.e., naïve sera).

### Neutralizing Antibody sVNT Assay.

Neutralization titers were determined using a GenScript SARS-CoV-2 sVNT Kit. Manufacturer’s instructions were followed. Briefly, negative Matrix Control (NMC) was made using mouse naïve serum diluted 10-fold with sample dilution buffer. A standard curve was generated using a monoclonal antibody purchased from the manufacturer (MAB, GenScript, A02051) which was first diluted to a final working concentration of 3 μg/mL with sterile water. A standard curve was created by diluting the MAB in NMC at the following concentrations: 300, 150, 75, 37.5, 18.75, 9.38, and 4.7 ng/mL. Samples from serum were diluted serially 1:3 starting at a 1:10 dilution and going down to a 1:810 dilution in sample dilution buffer. Positive and negative controls were provided by the manufacturer and used as instructed. HRP functionalized RBD (RBD-HRP) was made into a working solution following the manufacturer’s instructions. Upon preparation of all samples, a 96-well flat-bottom plate was obtained, and 120 μL of each standard curve dilution, 60 μL of each sample dilution, and 60 μL of each positive and negative control were added to individual wells. Immediately after, an equal volume of RBD-HRP working solution was added to each well, and the plate was incubated at 37 °C for 30 min. After incubation, 100 uL of these mixtures were immediately transferred to the manufacturer-provided ELISA plate precoated with ACE2 receptor, and the plate was sealed and incubated at 37 °C for 15 min. Wells were then washed four times with 1× wash solution (provided by kit), after which 100 μL of TMB solution was added to each well and incubated at RT for 15 min, protected from light. Timing was started after the addition of TMB to the first set of wells, as per manufacturer’s instructions. After 15 min, 50 uL of Stop Solution was added to each well in the exact same order as the TMB solution was added. Absorbance at 450 nm was immediately read using a BioTek Cytation 5.

### Proteomics Sample Preparation.

Total protein content in sera was quantified in order to compare relative amounts of proteins among different samples. To do this, a BCA assay was performed (Thermo-Fisher). Samples were sent to the Northwestern University Proteomics Core, where they were resuspended in 8 M urea, reduced with 4 mM dithiothreitol, and then alkylated with 18 mM iodoacetamide. The solution was then diluted to <2 M urea (final concentration), and trypsin was added at the final trypsin/protein ratio of 1:100 for overnight incubation at 37 °C. The resulting peptides were desalted using solid-phase extraction on a Pierce C18 Spin column. The eluates were dried under a vacuum and reconstituted with 5% ACN/0.1% formic acid (FA) in water.

### Mass Spectrometry Analysis and Database Search.

The obtained peptides were analyzed by liquid chromatography with tandem mass spectrometry (LC-MS/MS) using a Dionex UltiMate 3000 Rapid Separation nanoLC and a Q Exactive HF Hybrid Quadrupole-Orbitrap Mass Spectrometer (Thermo Fisher). Samples were loaded onto a house-packed C18 column and separated with a 5 to 40% gradient of solvent (0.1% FA in ACN) for 120 min by an analytical column (PicoChip, New Objective, Inc.). MS/MS spectra were searched against the SwissProt *Mus musculus* database using Mascot search engine (Matrix Science; version 2.7.0.1). All searches included carbamidomethyl Cys as a fixed modification and oxidized Met; deamidated Asn and Gln; and acetylated *N*-term as variable modifications. The search result was visualized by Scaffold v 5.0.1 (Proteome Software, Inc.). A 1% false discovery rate of the protein with a minimum of two unique peptides was identified. Statistical analysis, specifically, a Fisher’s exact test with a Benjamini–Hochberg multiple test correction, was performed for comparison between 200-SNA and 150-SNA groups (*n* = 4 and 3 samples per group, respectively).

### In Vivo Live Viral Challenge.

All work with live SARS-CoV-2 was performed safely in the BSL3 facility of the Ricketts Regional Biocontainment Laboratory, operated by the University of Chicago following a protocol approved by the IACUCs of both Northwestern University and the University of Chicago. The 6- to 8-wk-old female and male B6.Cg-Tg(K18-ACE2)2Prlmn/J (k18-hACE2) mice (Jackson) were challenged with 2 × 10^4^ pfu of USA-WA1/2020 SARS-CoV-2 (2019-nCoV) in 20 μL by intranasal injection. Mice were monitored twice daily to record clinical symptoms and weighed daily. Categories in clinical scoring can be found in *SI Appendix*, Table S3. Animals that lost 20% of their baseline body weight or had a clinical score of 3 were killed for humane reasons. Animals that did not meet these criteria were monitored for up to 12 d while in the BSL3 facility. On the day of sacrifice (either day 5 for PBS- or admix-treated groups or day 12 for SNA-treated groups), animals were killed and subjected to necropsy to remove the lungs. One part of the lungs was homogenized in 2% Dulbecco’s modified Eagle’s medium (DMEM) to measure viral titers (see *Quantification of Virus in Lung by Plaque Assay*), whereas the other part was fixed in paraffin-embedded blocks for histopathology.

### Lung Histopathology.

Formalin-fixed lung sections were embedded in paraffin blocks released from the BSL3 facility after verifying the absence of infectious virus and were used to generate slides for staining studies by the Mouse Histology & Phenotyping Laboratory, Northwestern University, as previously described (39). Histopathology was evaluated by two expert lung pathologists. Blinded lung pathologists evaluated the severity and presence of lung injury using a scoring system recently described in k18-hACE2 mice infected with SARS-CoV-2 (39, 40). The alterations scored were mononuclear infiltrates, neutrophils, edema, and necrosis. The scale was as follows: 0 = no detection, 1 = uncommon detection in <5% lung fields (∼200 Å), 2 = detectable in up to 30% of lung fields, 3 = detectable in 33 to 66% of lung fields, and 4 = detectable in >66% of lung fields. Neutrophil infiltration was evaluated on a scale of 0 to 3 as follows: 0 = within normal range, 1 = scattered polymorphonuclear leukocytes (PMNs) sequestered in septa, 2 = score 1 and solitary PMNs extravasated in airspaces, 3 = score 2 and aggregates in vessel and airspaces.

### Quantification of Virus in Lung by Plaque Assay.

Tissue samples were collected in DMEM containing 2% FBS and were homogenized with 1.4-mm ceramic beads in a tissue homogenizer using two 30-s pulses. Samples were subsequently centrifuged at 1,000 × *g* for 5 min, and the supernatant was collected and serially diluted 10-fold to infect VeroE6 cells. Cells were infected for 1 h, after which inoculum was removed, and 1.25% methylcellulose DMEM solution was added to the cells and incubated for 3 d. Plates were fixed in 1:10 formalin and stained with crystal violet for 1 h for counting to determine pfu per milliliter. Samples were normalized to milligrams of lung tissue, which was determined prior to homogenization.

### Statistical Analysis.

All values shown in graphs were mean ± SD or SEM, as described in each figure caption. Individual biological replicates are shown as points. Group sample size is described in each figure caption. Statistical analysis was performed using GraphPad Prism 9 software, and specific analysis is provided in each figure caption. Comparisons between two groups utilized an unpaired *t* test. Comparisons assessing more than two groups used an ANOVA with a post hoc test for multiple comparisons analysis between individual groups. Depending on whether the SD between groups could be assumed to be equivalent, either an ordinary one-way or a Brown–Forsythe ANOVA was used. Post hoc tests employed were either Sidak, Tukey, or Dunnett, depending on the assumptions based on SD differences. No specific preprocessing of data was performed prior to statistical analyses. Significance was defined as *P* < 0.05 (*P < 0.05; **P < 0.01; ***P < 0.001; ****P < 0.0001; ns = nonsignificant).

## Supplementary Material

Supplementary File

## Data Availability

All study data are included in the article and/or *SI Appendix*.
