# Supplementary material for: Spherical nucleic acids as an infectious disease vaccine platform
Source: Proc Natl Acad Sci U S A. 2022 Mar 21;119(14):e2119093119. doi: 10.1073/pnas.2119093119 (PMC9168922; doi:10.1073/pnas.2119093119)
Supplement: Supplementary File [file pnas.2119093119.sapp.pdf]

PNAS
www.pnas.org

Supplementary Information for
Spherical Nucleic Acids as an Infectious Disease Vaccine Platform

Michelle H. Teplensky[a,b,1], Max E. Distler [a,b,1], Caroline D. Kusmierz [a,b], Michael Evangelopoulos[c], Haley Gula[d], Derek Elli[d], Anastasia Tomatsidou[d], Vlad Nicolaescu[d], Ian Gelarden[e], Anjana Yeldandi[e], Daniel Batlle[f], Dominique Missiakas[d], and Chad A. Mirkin[a,b,c,2]

Chad Mirkin
Email: chadnano@northwestern.edu

This PDF file includes:

Figures S1 to S7
Tables S1 to S3

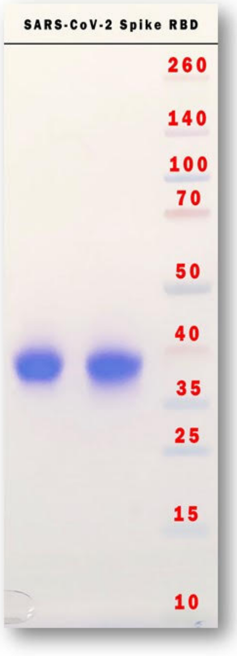

Fig. S1. SDS PAGE of SARS-CoV-2 Spike protein RBD. Protein was expressed in Expi293 and purified by Ni-affinity chromatography. Two different batches of sample are shown for demonstration of reproducible expression and purification. The molecular weight of the protein is 40,315.17 Da.

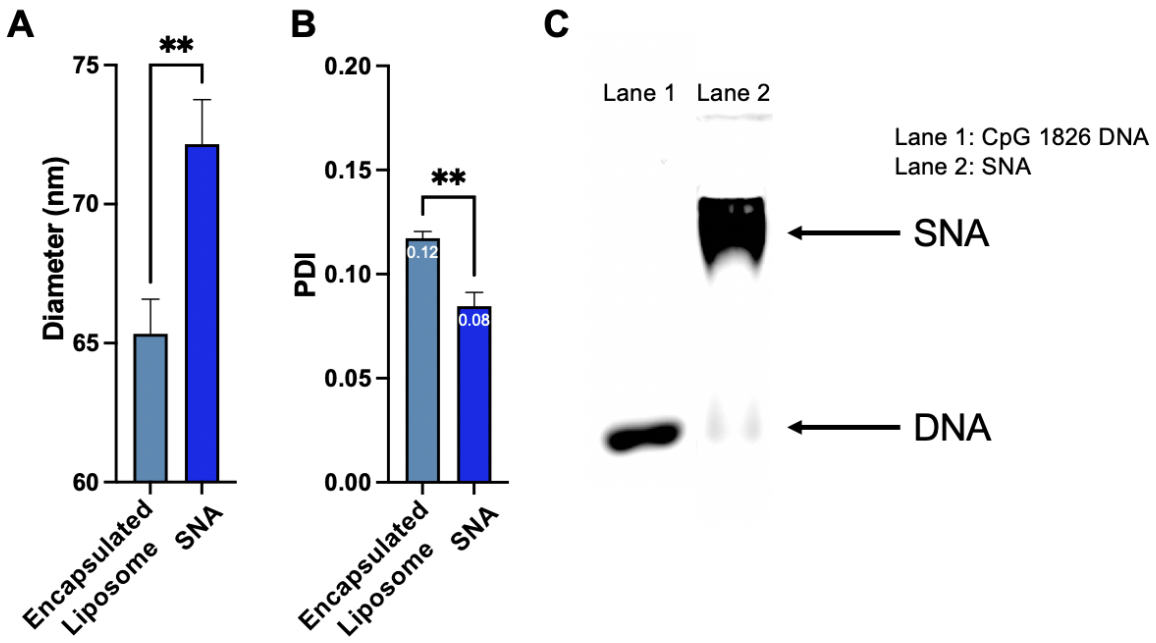

Fig. S2. SNA formation confirmed through dynamic light scattering (DLS) and agarose gel electrophoresis. (A) DLS demonstrates a significant increase in diameter and (B) a decrease in polydispersity index (PDI) as a result of addition of the DNA shell. (C) Agarose gel electrophoresis highlights the mobility shift of DNA as a result of SNA formation.

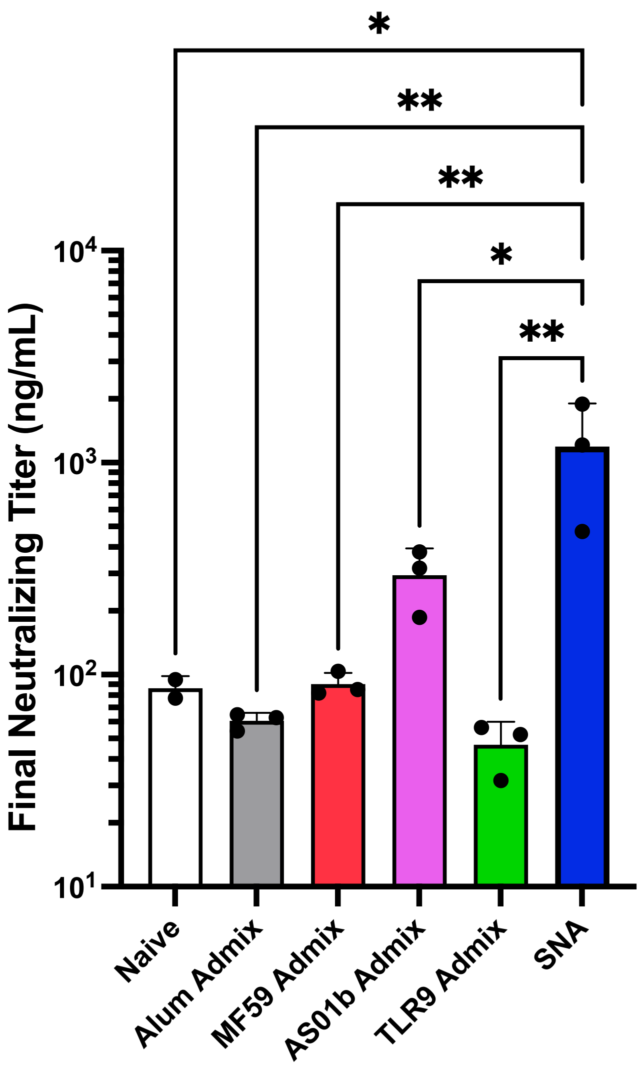

Fig. S3. Final neutralizing titer calculated in a pseudovirus inhibition study and fit to a standard curve using sera collected from C57BL/6 mice 21 days post a single prime injection. *p<0.05; **p<0.01.

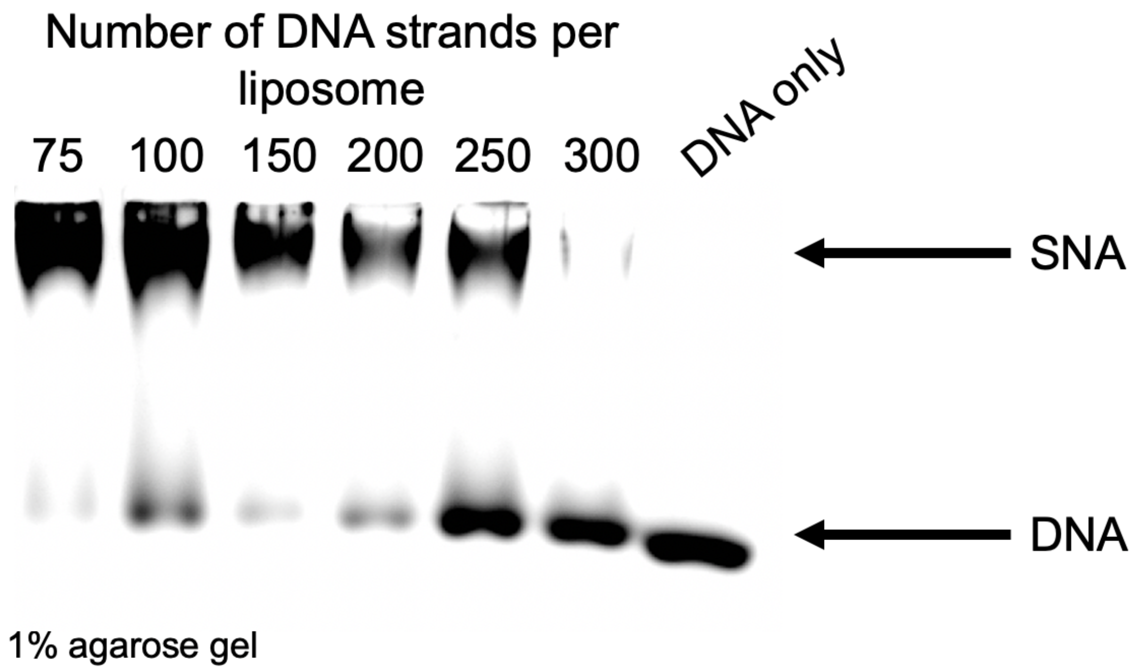

Fig. S4. Determination of maximum loading of DNA per liposome before dissociation. The last detectable SNA band that also has a low intensity DNA band, indicating low levels of dissociation, is present at a stoichiometry of 200 DNA strands per liposome.

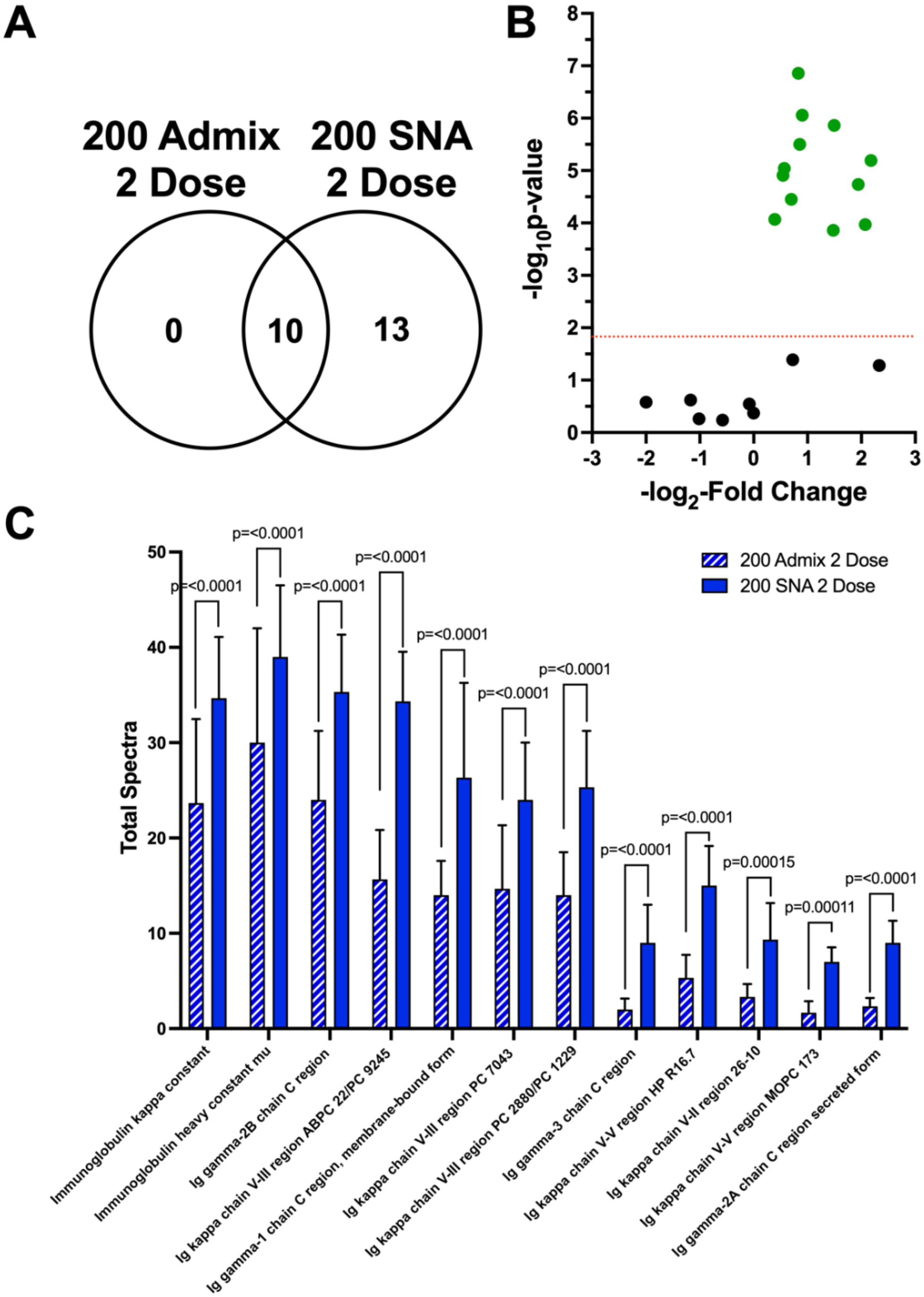

Fig. S5. (A) Quantitative profile of immunoglobulins between the 200 Admix 2 dose group and 200 SNA 2 dose group. (B) Volcano plot showing relative fold change and significance of different Igs when comparing the 200 Admix 2 dose against the 200 SNA 2 dose group. Red line indicates significance threshold. (C) The identified significant upregulated proteins were plotted as a function of total spectra with significance shown. Significance threshold = $p<0.01585$.

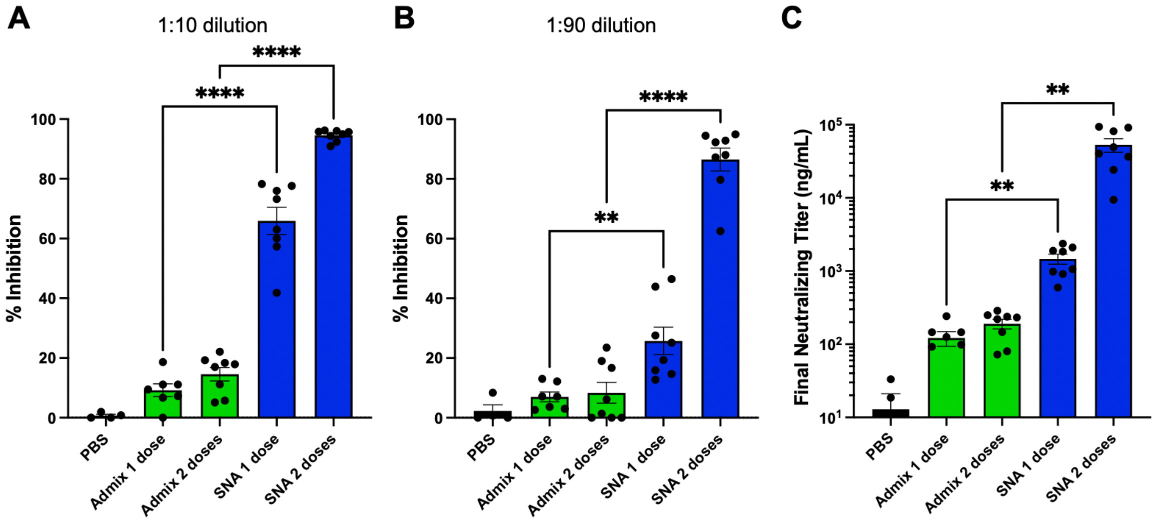

Fig. S6. Sera from k18-hACE2 transgenic mice were collected after immunization with different vaccines just prior to infection with virus to verify neutralizing antibody production. (A,B) Different dilutions of sera were assessed for antibody ability to inhibit RBD binding to ACE2 in a pseudovirus assay. SNA at either dose is significantly more effective at inhibition than admix. (C) Values from assay were fit to a standard curve to calculate a final neutralizing titer. **$p<0.01$; ****$p<0.0001$.

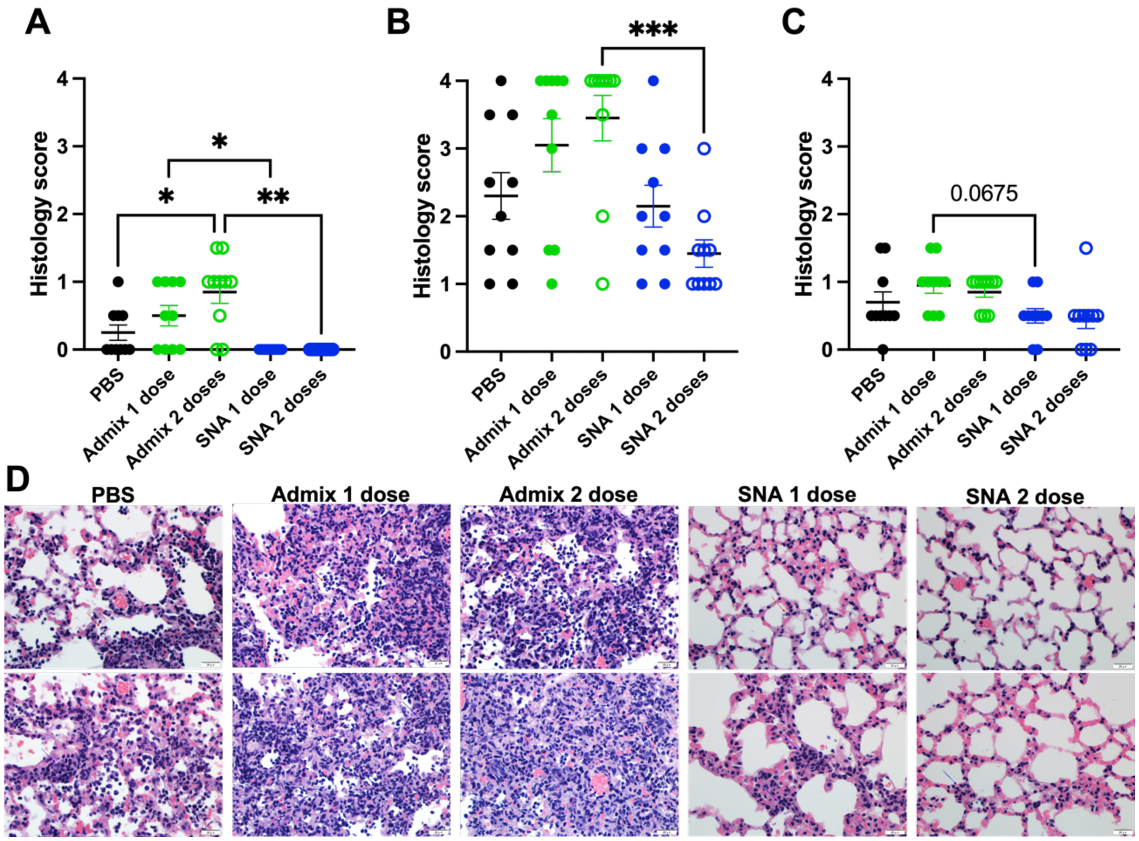

Fig. S7. Lung histopathology analysis from k18-hACE2 transgenic mice immunized with vaccines and challenged with a lethal infection of SARS-CoV-2. Lungs were collected on date of death (day 5 for PBS and admix vaccine either dose, and day 12 for SNA vaccine either dose). Scoring of (A) necrosis, (B) mononuclear infiltrates, and (C) edema. (D) Representative images of lungs stained with hematoxylin and eosin. Two representative images shown per group (magnification: 400x). Scale bar = 20 μm for all images. PBS group shows expansion of alveolar septae by inflammatory cells with solitary neutrophils in airspaces (red arrow, top image) and multiple foci of alveolar septal expansion by aggregates of mononuclear cells (blue arrows, bottom image). Admix 1 dose group shows dense inflammatory infiltrate including aggregates of neutrophils within airspaces (red arrow, top image) and diffuse expansion of alveolar septae by variably dense aggregates of mononuclear cells (blue arrows, bottom image). Admix 2 dose group shows dense inflammatory infiltrate including aggregates of neutrophils within airspaces (red arrow, top image) and diffuse mononuclear infiltrate consisting of variably sized lymphocytes with dense to open nuclear chromatin (blue arrows, bottom image) causing marked architectural distortion and expansion of alveolar septae. SNA 1 dose group shows rare, scattered neutrophils (red arrow, top image) sequestered in thin alveolar septae and foci of alveolar septal expansion by loose aggregates of mononuclear cells (blue arrows, bottom image). SNA 2 dose group shows thin alveolar septae with rare to absent neutrophils (top image) and focal expansion of alveolar septae by small loose aggregate of mononuclear cells (blue arrow, bottom image). $^{*}p<0.05$; $^{**}p<0.01$; $^{***}p<0.001$.

Table S1. DNA Adjuvant Sequences used in this work.

Name	Sequence (5' to 3')[3]	Backbone (PS = phosphorothioate)	Calc'd Mass g/mol	Extinction Coefficient L/(mole·cm)[1]
CpG 1826	TCC ATG ACG TTC CTG ACG TT $(Spacer18)_2$ Cholesterol	PS	6364.1	181100
CpG 1826 Cy5 Fluorophore -labeled[2]	TCC ATG ACG TTC CTG ACG TT Cy5 $(Spacer18)_2$ Cholesterol	PS	8405.6	191100
CpG 7909	TCG TCG TTT TGT CGT TTT GTC GTT $(Spacer18)_2$ Cholesterol	PS	7698.2	209400

[1] Calculated using IDT's OligoAnalyzer Tool: https://www.idtdna.com/calc/analyzer
[2] Cy5 = 1-[3-(4-monomethoxytrityloxy)propyl]-1'-[3-[(2-cyanoethyl)-(N,N-diisopropylphosphoramidityl]propyl]-3,3,3',3'-tetramethylindodicarbocyanine chloride; Stock no. 10-5915 (Glen Research)
[3]Spacer18 = 18-O-Dimethoxytritylhexaethyleneglycol,1-[(2-cyanoethyl)-(N,N-diisopropyl)]-phosphoramidite; Stock no. 10-1918 (Glen Research)

Table S2. List of Immunoglobulin (Ig) proteins detected by proteomics. Yellow shading indicates those that were significant in a Fisher's Exact T test between the 200 SNA and 150 SNA group.

Name	Molecular Weight	Fisher's Exact Test (p-value): *(p< 0.0063)[2]	Total Spectra Count (Integer avg for group)		
			75 SNA	150 SNA	200 SNA
Immunoglobulin kappa constant	12 kDa	0.37	4	37	26
Immunoglobulin heavy constant mu	50 kDa	< 0.00010	13	20	32
Ig gamma-2B chain C region	44 kDa	< 0.00010	6	12	19
Ig kappa chain V-III region ABPC 22/PC 9245	12 kDa	0.00016	7	12	17
Ig gamma-1 chain C region, membrane-bound form	43 kDa	0.059	4	14	14
Ig kappa chain V-III region PC 7043	12 kDa	0.00032	6	10	15
Ig kappa chain V-III region PC 2880/PC 1229	12 kDa	0.0011	0	11	15
Ig gamma-3 chain C region	44 kDa	0.17	2	7	6
Ig kappa chain V-V region HP R16.7	12 kDa	0.25	3	4	4
H-2 class I histocompatibility antigen, Q10 alpha chain	37 kDa	0.53	3	8	5
Ig kappa chain V-II region 26-10	12 kDa	0.079	1	4	5
Ig kappa chain V-V region MOPC 173	12 kDa	0.45	1	2	1
Ig heavy chain V region AC38 205.12	13 kDa	0.4	0	3	3
Ig gamma-2A chain C region secreted form	37 kDa	0.32	2	2	2
Ig heavy chain V region 93G7	16 kDa	0.56	0	1	1
Ig heavy chain V-III region HPC76 (Fragment)	12 kDa	0.086	0	0	1
Ig kappa chain V-V region MOPC 41	14 kDa	0.18	0	1	1
Ig heavy chain V region HPCG14	14 kDa	0.29	0	1	1
Polymeric immunoglobulin receptor	85 kDa	0.47	0	0	0
Ig alpha chain C region	37 kDa	1	0	0	0

Table S3. Clinical Scoring Table for *In Vivo* Lethal Viral Challenge Study

Clinical Score	Description
0	(Pre-Inoculation) - Mice Are Bright, Alert, Active, Normal Fur Coat and Posture.
1	(Post-Inoculation) - Mice Are Bright, Alert, Active, Normal Fur Coat and Posture, No Weight Loss.
1.5	(Post-Inoculation) - Mice Present with Slightly Ruffled Fur but Are Active **OR** Weight Loss Might Occur But Does Not Reach 2.5%; Recovery Can Be Expected.
2	(Post-Inoculation) - Ruffled Fur **OR** Less Active **OR** < 5% Weight Loss; Recovery Might Occur.
2.5	(Post-Inoculation) - Ruffled Fur **OR** Not Active but Moves When Touched OR Hunched Posture **OR** Difficulty Breathing **OR** Weight Loss 5-10%; Recovery Is Unlikely But Still Might Occur
3	(Post-Inoculation) - Ruffled Fur **OR** Inactive but Moves When Touched **OR** Difficulty Breathing **OR** Weight Loss At 11- 20%; Recovery Is Not Expected
4	(Post-Inoculation) - Ruffled Fur OR Positioned on Its Side or Back **OR** Dehydrated **OR** Difficulty Breathing **OR** Weight Loss >20% **OR** Labored Breathing; Recovery Is Not Expected
5	(Post-Inoculation) - Death
